# Conditional Senescence and Longevity Mechanisms in Early-Branching Metazoans: Insights from *Hydra*

**DOI:** 10.3390/cimb48090956

**Published:** 2026-09-19

**Authors:** Valeria Russo, Ion Udroiu, Valentina Cianfanelli, Veronica D’Ezio, Riccardo Proietti, Antonella Sgura, Tiziana Persichini, Marco Colasanti

**Affiliations:** Department of Science, Roma Tre University, 00146 Rome, Italy

**Keywords:** autophagy, *Hydra*, microbiome, nitric oxide signaling, oxidative stress, telomerase, transient receptor potential channel

## Abstract

Organismal aging is not inevitable in multicellular animals, as early-branching metazoan lineages, such as cnidarians, display negligible senescence under defined conditions, despite conserved cellular pathways. Contrasting longevity phenotypes in *Hydra* species reveal distinct regulatory mechanisms underlying aging. A comparison between *Hydra vulgaris* and *Hydra oligactis* suggests that sustained telomerase activity, autophagy regulation, and microbiome stability in *H. vulgaris* are associated with stem cell renewal and long-term tissue homeostasis, whereas cold-induced stress in *H. oligactis* is accompanied by a disruption of these pathways, leading to rapid somatic decline, where autophagy dysfunction and microbiome dysbiosis may act as contributing or amplifying factors. Environmental sensing via conserved pathways may integrate these regulatory mechanisms. These observations suggest that aging in early-branching metazoans is a regulated, context-dependent process characterized by substantial lineage-specific variation rather than an inevitable, universal consequence of cellular senescence. Further mechanistic studies may provide evolutionary insights into longevity mechanisms and identify potential targets for modulating aging, although direct experimental validation remains necessary.

## 1. Introduction

Classical evolutionary theory posits that senescence is an inevitable consequence of multicellularity. However, contemporary research distinguishes organismal aging, characterized by age-dependent deterioration, declining vitality, and increased mortality, from cellular senescence, a permanent state of cell cycle arrest with a senescence-associated secretory phenotype (SASP) [1,2,3]. These processes differ in terms of their mechanisms and evolution. Cellular senescence is conserved across vertebrates and invertebrates and plays an adaptive role in development, tissue repair, and tumor suppression [1,4]. In contrast, organismal aging involves a coordinated decline across multiple systems that varies substantially among lineages. We propose an interpretative hypothesis that organismal aging, rather than cellular senescence pathways, represents a derived character. This model suggests that the coupling of cellular stress/senescence pathways to progressive, irreversible organismal decline has evolved selectively in certain complex lineages, rather than representing an obligatory program across all multicellular organisms.

Comparative phylogenomic analyses have revealed that organismal aging is a derived condition that evolved independently in specific lineages [5,6,7]. Early-branching animals, such as sponges, placozoans, ctenophores, and cnidarians, possess remarkable longevity and regenerative capacity, supported by abundant pluripotent stem cells [8]. Despite conserved cellular senescence pathways, basal animals exhibit minimal organismal senescence, characterized by extremely low mortality rates and indefinite regenerative capacity [9]. Conserved cellular senescence pathways do not automatically translate into progressive organismal aging. Early-branching metazoan lineages with indeterminate growth appear to limit the accumulation of cells displaying senescence-like states. It is critical to note that bona fide cellular senescence, traditionally defined in mammalian models by stable cell cycle arrest, senescence-associated β-galactosidase (SA-β-gal) expression, and a specific SASP, has not been molecularly validated or characterized in cnidarians [2,6,10,11,12,13]. Therefore, senescence-like cellular states and stress pathways must be strictly distinguished from whole-organism or demographic aging to prevent the unwarranted extrapolation of conserved stress pathways. This aligns with the molecular ‘hallmarks of aging’, including telomere attrition, DNA damage, mitochondrial dysfunction, impaired macroautophagy, stem cell exhaustion, chronic inflammation, and dysbiosis [14,15].

Aging may represent a regulated outcome shaped by evolutionary context, tissue organization, and ecological constraints [16]. Senescence is coupled to organismal aging only in certain lineages, particularly large, long-lived vertebrates and terrestrial organisms, where tissue complexity and cancer pressure favor obligatory aging programs [6,17,18]. In contrast, in early-branching metazoans, senescence is highly plastic and conditional, with its manifestation influenced by energetic trade-offs and specific eco-physiological responses to environmental conditions. To ensure a transparent, balanced, and evidence-based synthesis for this perspective, we conducted structured literature searches until June 2026. Primary peer-reviewed studies were prioritized for inclusion. Cutting-edge preprints and repository records (including Zenodo deposits) were screened and incorporated to present the newest insights but were explicitly identified as preliminary or non-peer-reviewed in the text to maintain analytical balance.

## 2. Organismal Aging as a Derived Character in Early-Branching Metazoans

Porifera, exemplified by *Oscarella lobularis*, display remarkable regenerative capacity through tissue remodeling and cell transdifferentiation [19,20,21]. Organism coloniality may explain senescence patterns: *Hydractinia echinata*, a colonial cnidarian, exhibits a more rapid age-related decline than solitary *Hydra* species [17], suggesting that organismal integration correlates with senescence risk. Placozoa (*Trichoplax adhaerens*) display minimal tissue organization and reproduce asexually via fission [22]. Simple tissue organization and macrophage-like fiber cells phagocytosing damaged cells may limit the accumulation of dysfunctional cells and provide cellular quality control [22]. Ctenophora maintain active stem cell niches where germline-associated genes (*vasa*, *piwi*, *and nanos*) mark proliferative cells [5,23]. *Pleurobrachia pileus* and *Mnemiopsis leidyi* show somatic stem cell niches, with vasa/piwi marking non-germline proliferative populations [24,25,26]. The Germline Multipotency Program (GMP) proposes that this gene set represents an ancestral multipotency toolkit predating strict soma-germ segregation [25], although direct functional cooperation should be considered an interpretative model rather than an established mechanistic fact.

Cnidaria, particularly *Hydra*, is a crucial aging model [27,28] because this lineage escapes senescence through continuous stem cell self-renewal [29]. Three independent *Hydra* stem cell lineages maintain indefinite self-renewal capacity, powering continuous asexual reproduction and regeneration [30]. Demographic studies on *Hydra vulgaris* have documented extremely low mortality rates and no detectable decline in asexual reproductive output over four years [31,32], consistent with negligible senescence under controlled conditions. However, apparent negligible senescence in wild populations may reflect sampling artifacts [33], suggesting that ‘biological immortality’ should be interpreted as negligible senescence within experimentally tractable settings.

In contrast, *Hydra oligactis* exhibits conditional and environment-induced senescence, which is strictly strain-specific [34,35,36]. Specifically, these phenotypes occur in cold-sensitive strains of *H. oligactis* (HoCS), whereas cold-resistant strains (HoCR) maintain homeostasis at identical temperatures. At approximately 18 °C, cold-sensitive *H. oligactis* proliferates asexually. At 10 °C, prolonged thermal stress in these sensitive strains triggers sexual reproduction, stem cell pool depletion, and a rapid, coordinated decline. This aging-like deterioration is characterized by epithelial cell vacuolization, bodily shrinkage, and loss of regenerative capacity, which are demographic and organismal phenotypes that should not be conflated with marker-defined cellular senescence. Cold-induced senescence in *H. oligactis* may result from either direct cellular toxicity or from an integrated developmental program in which cold sensing triggers a switch from asexual to sexual reproduction, a scenario described as the “polyphenism model”. In this model, senescence is viewed as an active developmental decision, where the organism prioritizes reproductive output over maintenance in response to environmental cues [17]. Comparative studies of cnidarians that undergo reproductive switching support the idea that the polyphenism model may better explain the observed patterns of senescence [37]. However, it remains unclear whether early-branching metazoans possess a conserved program that coordinates reproductive and somatic trade-offs in a manner distinct from that observed in classical model organisms, highlighting an important gap in our understanding.

## 3. Telomerase Expression and Senescence

Telomeres are nucleoprotein complexes that protect chromosome ends and exhibit profound evolutionary conservation. The TTAGGG hexanucleotide repeat emerged early in eukaryotic evolution and is highly conserved in all animals, except for Arthropoda and Nematoda. Similarly, the architecture of the Shelterin complex is highly conserved in all animals (with the notable exception of Diptera), showing just duplication of some proteins in some vertebrate lineages [38]. The “end replication problem”, partial loss of terminal telomeric sequences at each cell division [39], causes progressive telomere shortening in somatic cells, triggering replicative senescence or apoptosis when the length falls below a critical limit [40,41]. Telomerase reverse transcriptase (TERT) counterbalances this attrition by adding DNA repeats to the chromosome extremities [12,42,43]. Across choanoflagellates, porifers, cnidarians, and vertebrates, TERT adds the conserved TTAGGG motif, whereas it diverges in nematodes and arthropods [34,38,44].

Organismal negligible senescence may require constitutively active telomerase in somatic tissues to prevent senescent cell accumulation and limit inflammaging [12]. High telomerase activity in marine demosponges and asexual planarians, both of which exhibit minimal aging and robust regeneration, supports the hypothesis linking low senescence to elevated TERT activity in somatic populations [45,46]. TERT from *Aurelia aurita* retains catalytic activity [44], and transcriptomic analysis of *Hydra* revealed substantial TERT expression in stem cells compared to differentiated cells [47]. Moreover, TERT expression and telomerase activity were recently demonstrated in *H. vulgaris*, being higher in buds and body regions than in the foot and head [48]. Another recent preliminary study on *H. vulgaris* confirmed the presence of telomerase activity [49]. In these experiments, chronic treatment with epigallocatechin gallate (EGCG), a pharmacological compound known to exert pleiotropic effects on multiple cellular targets and redox pathways, was associated with a reduction in telomerase activity and impaired regenerative capacity after 2–3 weeks [49]. This pharmacological link suggests a correlation but cannot be considered definitive evidence that active telomerase is essential. Genetic loss-of-function validation or rescue experiments are necessary to establish target-specific causality. We must explicitly separate the distinct nodes of this pathway: TERT expression, telomere-length maintenance by telomerase activity, regenerative capacity, and organismal longevity. In *Hydra*, TERT expression and catalytic activity have been detected; however, direct functional evidence linking telomerase activity to organismal longevity is currently lacking and remains a major unresolved question. Whether constitutive telomerase activity causally sustains indefinite somatic maintenance or represents a permissive background feature requires future genetic loss-of-function models and longitudinal telomere-length dynamics. Direct, longitudinal telomere-length measurements are virtually absent from the literature. Furthermore, the 2026 Zenodo reference [49] represents a preliminary, non-peer-reviewed repository. Direct genetic loss-of-function models for TERT have not yet been established in cnidarians. Elevated TERT levels in stem cells suggest an enhanced capacity to counteract telomere attrition. Senescence in *H. oligactis* occurs only after cold exposure, suggesting stress-induced rather than replication-driven senescence. Additionally, recently reported telomerase data in *Hydra* highlight unresolved questions. Available analyses suggest that telomerase activity in *Hydra* stem cell compartments is constitutively high and remains elevated during head regeneration, rather than showing a strong regeneration-specific upregulation [34]. Representing both areas of agreement (TERT stem cell enrichment) and disagreement (regeneration-specific induction kinetics) is essential for a balanced evolutionary perspective, emphasizing that the exact dynamics of regeneration-associated TERT remain under active scientific debate.

## 4. Autophagy and Microbiome Stability as Coordinators of Longevity

### 4.1. Autophagic Flux and ULK1-Dependent Somatic Homeostasis

*Hydra* provides a sharp contrast between the sustained low mortality and lack of overt age-associated decline observed in fed, asexual *H. vulgaris*, in which continuous stem-cell self-renewal supports long-term tissue homeostasis, and the rapid inducible aging phenotype of HoCS following cold-induced gametogenesis [34,35,36]. In HoCS, this phenotype includes the loss of somatic interstitial cells, reduced epithelial cell proliferation, impaired regeneration, behavioral decline, and death within a few months. By contrast, HoCR animals either remain asexual or transiently undergo gametogenesis and subsequently recover physiological fitness without progressive deterioration [34,35,36]. These distinct outcomes correlate with differences in the capacity of epithelial cells to induce autophagic flux. Autophagy is a major proteostatic and metabolic pathway that couples nutrient and stress sensing to lysosomal degradation and recycling of cytoplasmic components, including damaged proteins and organelles [50,51,52].

In *H. vulgaris*, starvation induces autophagy in epithelial cells, as indicated by the formation of LC3-positive autophagic structures and increased LC3-II levels. Conversely, RNA interference (RNAi)-mediated depletion of WIPI2, a factor required for LC3 lipidation, impairs autophagy in *H. vulgaris* and induces a phenotype resembling the deterioration observed in aging HoCS animals. WIPI2-depleted polyps displayed reduced body size, apical epithelial disorganization, increased levels of autophagy substrate, fewer LC3 puncta, impaired head regeneration, and reduced survival. These findings support a role for epithelial autophagic flux in cellular quality control, tissue homeostasis, and regenerative competence [36,53,54].

Interestingly, autophagy is differentially regulated in HoCS and HoCR strains of *H. oligactis*. HoCS epithelial cells exhibit defective autophagy inducibility, as evidenced by limited LC3-positive structure formation, p62/SQSTM1 accumulation, altered responses of an in vivo autophagy flux reporter, and poor response to starvation, proteasome inhibition, and rapamycin. Therefore, the defect is not restricted to cold exposure but is apparent under basal conditions and in response to several autophagy-modulating stimuli. Notably, HoCS and HoCR also differ constitutively in other proteostatic properties, including proteasome tolerance and Hsp70 processing, indicating that the autophagy defect is part of a broader, strain-specific difference in proteostatic capacity rather than an isolated lesion. Nevertheless, cold transfer provides the physiological context in which this defect becomes particularly consequential: gametogenesis-associated loss of somatic interstitial cells is followed by persistent loss of epithelial proliferation in HoCS, whereas epithelial cycling is restored in HoCR [36].

These observations are consistent with the hypothesis that deficient epithelial autophagy restricts the capacity of HoCS animals to adapt to interstitial cell loss and the associated nutritional and physiological challenges. However, the available evidence does not establish autophagy impairment as the initiating cause of cold-induced aging. It remains unclear whether the distinct autophagy phenotypes of HoCS and HoCR reflect intrinsic strain-specific differences in autophagy regulation, differential sensitivity to interstitial cell loss, or additional upstream consequences of cold-induced gametogenesis. Direct genetic manipulation of autophagy genes in HoCS and HoCR, combined with longitudinal analyses of epithelial proliferation, regeneration, behavior, and survival, will be required to test causality [36].

ULK1 is a conserved serine/threonine kinase that promotes autophagosome initiation. In *Hydra*, a ULK1/2-related gene and other components of the ULK1 are conserved and expressed, particularly in epithelial stem cells. In vitro kinase assays using recombinant human ULK1 showed that extracts from *H. vulgaris* enhanced human ULK1 activity, whereas extracts from fed HoCR and, more strongly, HoCS animals repressed it. These experiments identified differential activities in *Hydra* extracts that modulate mammalian ULK1 in vitro, but the responsible endogenous factor(s) remain unknown [55]. Because *Hydra* strains also differ in size, drug sensitivity, and reproductive mode, a systematic comparison of ULK1 regulation across additional *H. vulgaris* and *H. oligactis* strains will be needed to determine how much of this repression is specific to HoCS versus shared among *H. oligactis* strains.

Functional experiments nevertheless support a role for ULK1 in *Hydra* homeostasis and regeneration. Pharmacological inhibition with SBI-0206965 impaired apical regeneration, reduced contractility, and decreased body size in *H. vulgaris*. Repeated ULK1 RNAi similarly reduced animal size and contractility, although it did not significantly impair apical regeneration, likely reflecting the transient nature of RNAi-mediated knockdown; together, these results support a requirement for ULK1 activity in maintaining autophagy, regenerative competence, and organismal fitness, with its role in regeneration established pharmacologically rather than genetically in this system. However, because effective SBI-0206965 concentrations were relatively high and induced toxicity in HoCS animals, SBI-0206965 may have off-target activities and the molecular mechanism underlying ULK1 regulation in HoCS remains unresolved. The conclusion should remain that ULK1 contributes to autophagy-dependent homeostasis in Hydra, rather than that differential ULK1 activity alone explains the HoCS aging phenotype [55].

These data align with evidence that functional autophagy supports longevity across yeast, worms, flies and mice [56,57,58,59]. It also aligns with autophagy being critical for maintaining stem-like, non-senescent states in cancer stem cells and regulating the balance between stemness and differentiation fate [60,61].

Beyond these specific signaling differences, *Hydra* data align with a broader view of aging as an emergent consequence of stochastic molecular damage that accumulates when maintenance and repair systems are insufficient rather than as the output of a dedicated aging program. In this framework, autophagy constitutes a central maintenance layer that continuously clears damaged proteins and organelles, thereby limiting proteotoxic stress and preserving metabolic fitness in epithelial stem cells [36,55,57,58]. Sustained ULK1-dependent autophagic flux in *H. vulgaris* may thus narrow the gap between damage production and repair, keeping the cumulative burden of molecular errors below the threshold required for organismal decline, whereas cold-induced autophagy suppression in *H. oligactis* may widen this gap and permit damage and noise to propagate into rapid senescence [34,35].

### 4.2. Host–Microbiome Dynamics and Evolutionary Reprogramming Trade-Offs

In addition to autophagic clearance, somatic integrity in early-branching metazoans is closely linked to epithelial barrier functions and host–microbiome interactions. To avoid overintegrating these cellular modules, we must separate the direct evidence: (i) FoxO plays an established, direct functional role in stem cell self-renewal and maintenance; (ii) FoxO regulates microbiome composition by driving the expression of antimicrobial peptides; and (iii) autophagy is required for tissue homeostasis. A direct, causal bridge connecting FoxO-mediated microbiome control to autophagic competence remains hypothetical [62,63]. Furthermore, the available evidence is significantly stronger for host genetic control of microbiome composition than for the microbiome actively driving aging. Until germ-free, bacterial-depletion, or reconstitution experiments establish directionality, microbiome dysbiosis under cold stress should be consistently described as an associated or potentially amplifying factor of senescence rather than a demonstrated primary cause [30,62]. Similar bidirectional links between dysbiosis and immune dysfunction occur in vertebrates, although causal direction remains incompletely resolved [64,65,66].

This challenge of resolving causal hierarchies extends to two seemingly distinct explanatory frameworks for cold-induced senescence in HoCS: (1) the active developmental model (reproductive switching/polyphenism) and (2) the stochastic damage accumulation model [35,67]. These frameworks can be vertically integrated into a single unified cascade. We propose that cold-induced activation of gametogenesis represents an upstream evolutionary switch. Under cold stress, FoxO-mediated transcriptional reprogramming is hypothesized to repress somatic maintenance genes and prioritizes sexual reproduction, potentially leading to a systemic reallocation of resources that downregulates downstream maintenance systems, including autophagic flux and epithelial immune defense. This integrative model is supported by the observation that, while HoCS epithelial cells constitutively repress ULK1 activity, the upstream factors driving this inhibition remain uncharacterized. Consequently, stochastic molecular damage (such as proteotoxicity and mitochondrial dysfunction) and microbiome dysbiosis accumulate rapidly because the repair and clearance systems are turned down. Thus, organismal senescence is an emergent, stochastic consequence of an upstream, regulated developmental decision. Experimental decoupling is required to test this cascade and isolate direct cold toxicity from reproductive-driven somatic downregulation. The precise causal and temporal ordering among stem-cell depletion, autophagy impairment, and microbiome alterations during this cold-induced decline remains experimentally unresolved.

## 5. Environmental Sensing and Stress Integration: A Hypothetical Role for TRP Channels and Nitro-Oxidative Stress

Early-branching metazoans integrate environmental stimuli through highly conserved signaling pathways, with thermal danger generally mediated by the Transient Receptor Potential (TRP) superfamily of ion channels [68,69,70]. In *Caenorhabditis elegans*, the thermosensitive TRPA1 channel has been shown to modulate longevity through FoxO-dependent pathways [71]. In *Hydra*, FoxO is a critical regulator of stem cell maintenance [72], but a direct functional TRPA1-FoxO pathway remains undocumented. We propose a testable hypothesis that in *Hydra*, TRP-dependent environmental sensing may function as an upstream sensory layer that interfaces with FoxO-governed homeostatic programs. However, this interaction remains speculative in cnidarians and must be rigorously distinguished from cross-species analogies.

Genetic analyses of *Hydra magnipapillata* have revealed TRP channel expansion (approximately four TRPA, three TRPM, and five TRPVL channels), suggesting early diversification of sensory functions [73]. In *H. vulgaris*, TRPA1 activation by cold exposure promotes nuclear translocation of Nrf2 and NF-κB, driving the expression of superoxide dismutase (SOD) and nitric oxide synthase (NOS), and the secretion of ancestral antimicrobial peptides, including hydramacin and periculin [74]. Cold-induced TRP activation may similarly trigger these pathways in *H. oligactis*, potentially contributing to microbiome dysbiosis and Wnt/β-catenin disruption. Differential TRPA1-mediated cold responses between strains may represent a functional switch between negligible senescence and rapid aging.

TRPM3 activation by noxious heat leads to nitric oxide (NO) production and oxidative pathways, including Nrf2, SOD, and HSP70 [75]. NO operates through “three-dimensional multiplexing” based on temporal, spatial, and concentration-dependent coordinates [76,77]. At nanomolar concentrations, NO acts as a developmental regulator and cytoprotective messenger. During apical regeneration in *H. vulgaris*, localized NO activates the sGC–cGMP-protein kinase G cascade, which is required for head specification and blastema differentiation [76,78]. Basal NO levels may contribute to microbiome stability and stem cell niche function, whereas high NO levels drive irreversible post-translational modifications and peroxynitrite formation in the presence of reactive oxygen species (ROS), causing persistent damage [76]. In mammalian cell lines, excessive nitro-oxidative stress has been shown to modify redox-sensitive autophagy proteins (such as ATG4 or ULK1) via post-translational *S*-nitrosylation and nitration, leading to functional dysregulation [79,80,81,82,83,84]. In *Hydra*, while heat-induced TRPM3 activation drives NO production and oxidative stress responses [75], a direct biochemical link between nitro-oxidative modifications and autophagy dysregulation has not yet been tested. We hypothesize that cold- or heat-induced nitro-oxidative stress in *Hydra* may post-translationally inhibit ATG4 or ULK1, representing a testable biochemical mechanism for the observed autophagy block.

Redox signaling converges with autophagy to modulate lifespan, as catalase-mediated shifts in hydrogen peroxide levels can promote pro-longevity states that depend on intact autophagy and redox-sensitive ATG4 and ULK1 regulation [79,82,83,85]. Thus, autophagy sits at the intersection of proteostasis and redox homeostasis, and differential control of autophagic flux in *Hydra* strains distinguishes those that maintain negligible senescence from those that undergo aging, although the respective contributions of genomic maintenance, endoplasmic reticulum proteostasis, and nutrient sensing remain unquantified [34,36,55,57].

In mammalian systems, TERT exhibits non-canonical, extra-telomeric functions, including localization to the mitochondria to protect mtDNA, reduce ROS, and interact with Wnt/β-catenin and NF-κB signaling [86,87,88,89,90,91]. We hypothesize that *Hydra* TERT might possess similar non-canonical, extra-telomeric roles that interface with the autophagy–redox axis during regeneration. However, this non-canonical TERT-redox link in *Hydra* remains speculative and represents an open area for future biochemical investigations (the respective evidence levels of these interactions are classified in Table 1).

## 6. Conclusions

This perspective suggests that cellular senescence pathways do not inevitably drive organismal aging. Early-branching metazoans possess senescence components homologous to those in mammals but exhibit minimal aging, indicating that senescence operates as conditional rather than obligate. In *H. vulgaris*, constitutive telomerase activity in stem cells, microbiome control, and sustained autophagic flux maintain negligible senescence and an indefinite regenerative capacity. In contrast, cold stress in *H. oligactis* is associated with rapid somatic decline accompanied by simultaneous disruption of maintenance systems, where FoxO-dependent transcriptional reprogramming is hypothesized to shift metabolism toward sexual reproduction, coinciding with autophagy failure and microbiome dysbiosis.

Autophagy is a well-documented correlate of negligible senescence. Pharmacological inhibition in *H. vulgaris* produces aging-like deterioration, and differential ULK1 activity distinguishes cold-resistant from cold-sensitive strains.

The proposed integrative model links hypothetical TRP-mediated environmental sensing, nitric oxide signaling, oxidative stress, FoxO coordination, and microbiome homeostasis, acting as an associated or amplifying factor, with autophagy as the central maintenance process that may integrate these cues.

**Figure 1:**
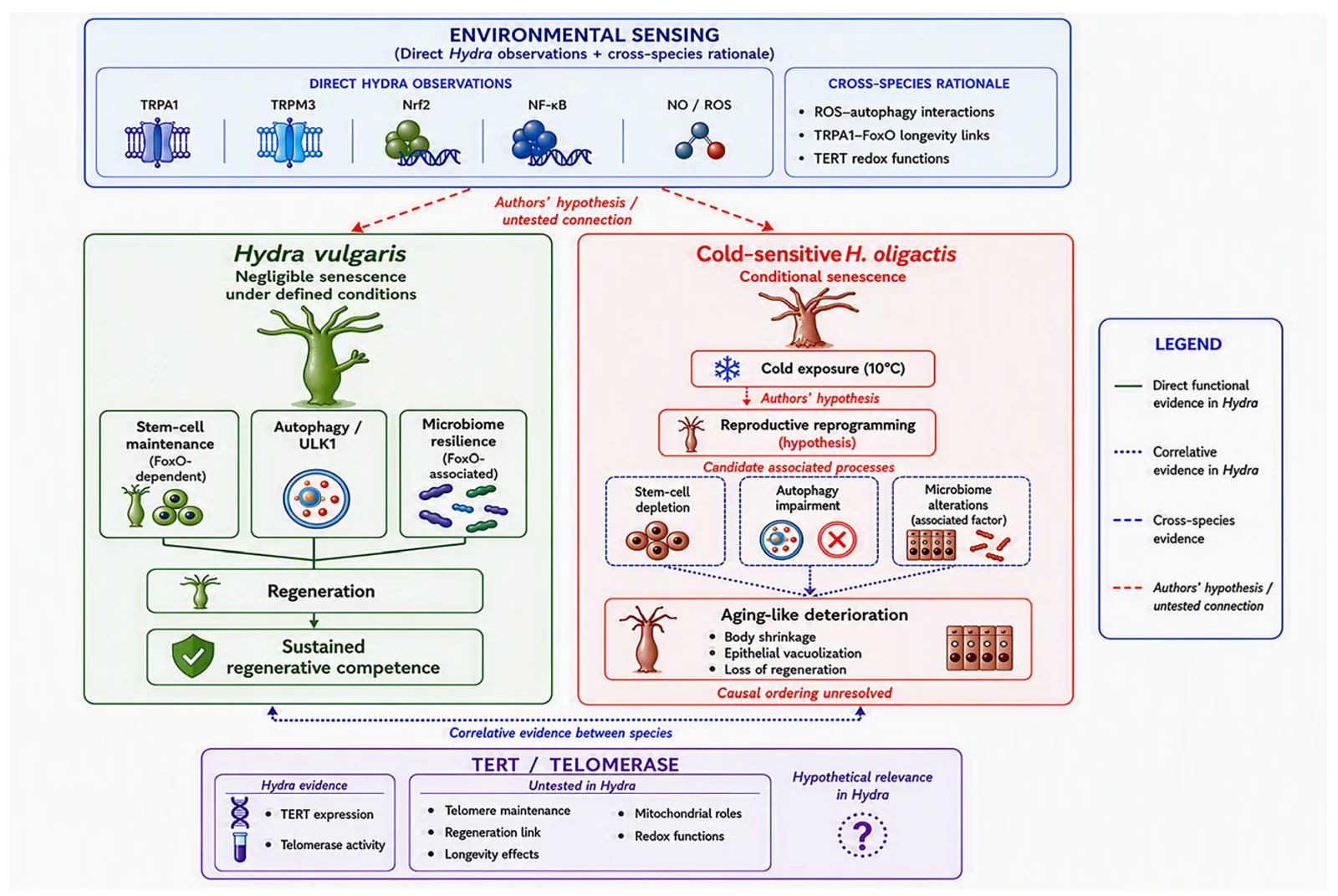
Evidence-based framework for negligible and conditional senescence in *Hydra*. In *H. vulgaris* (left, green panel), FoxO-associated stem-cell maintenance, autophagy/ULK1-supported homeostasis, and microbiome resilience are linked to sustained regeneration and long-term regenerative competence under defined laboratory conditions. I n cold-sensitive *H. oligactis* (right, red panel), cold exposure (10 °C) is associated with reproductive reprogramming (authors’ hypothesis), alongside stem-cell depletion, autophagy impairment, and microbiome alterations, which are characterized as contributing or amplifying factors rather than proven primary drivers. These processes are represented as correlative observations in *Hydra* and should not be interpreted as a demonstrated causal sequence; their temporal and mechanistic relationships remain unresolved. The environmental-sensing module (top, blue panel) presents TRP-mediated pathways strictly as testable hypotheses, distinguishing direct *Hydra* observations (TRPA1, TRPM, Nrf2, NF-κB, and NO/ROS signaling) from cross-species evidence and analogies that motivate, but do not establish, the proposed upstream regulatory connections. The TERT/telomerase module (bottom, purple panel) separates direct *Hydra* evidence (TERT expression and telomerase activity) from functions that remain untested in cnidarians, including telomere maintenance, regeneration-related effects, longevity associations, and mitochondrial or redox-related roles. Comparative evidence is included only as contextual support and should not be interpreted as evidence of conserved pathway wiring or physiological functions in *Hydra*. Line styles correspond to the evidence categories defined in the legend: solid green, direct functional evidence in *Hydra*; dotted blue, correlative evidence in *Hydra*; dashed blue, cross-species evidence; dashed red, authors’ hypotheses or untested connections.

Although individual components operate in *Hydra*, direct evidence of a unified pathway function is lacking (see Figure 1 for a schematic of the proposed integrative model).

To resolve the causal directionality of microbiome dysbiosis, future experiments must utilize targeted microbiota manipulation. Cultivating germ-free cold-sensitive *H. oligactis* polyps, followed by selective bacterial depletion or defined-community reconstitution, will determine whether maintaining eubiotic microbiota can prevent or rescue cold-induced somatic decline or whether dysbiosis is a secondary consequence of host tissue collapse. Specifically, (i) if germ-free animals senesce faster under cold stress, the microbiome acts as an amplifier; (ii) if reconstitution prevents the decline, the microbiome is a primary driver; and (iii) if reconstitution has no effect, the decline is a downstream consequence of host somatic failure. In parallel, it will be important to determine whether *Hydra* possesses mitochondrial TERT, potentially linking redox protection to autophagic regulation and FoxO signaling, which would suggest an evolutionary repurposing of telomerase as a coordinator of senescence suppression, although this remains speculative. Together, these lines of investigation will clarify whether organismal senescence in early-branching metazoans represent a regulated, conditional outcome of maintenance system failure rather than an inevitable consequence of damage accumulation, with broader implications for aging control and longevity pathways across animals.

Additionally, testing whether pharmacological autophagy induction (using rapamycin or trehalose) can rescue the aging phenotype in cold-sensitive strains under cold stress is a crucial experimental step. However, caution must be exercised when using these drugs, as rapamycin has broad effects on cell growth and translation, and trehalose can act as an osmotic protector and chemical chaperone. Therefore, any observed phenotypic rescue must be paired with pathway-specific biochemical readouts (such as measuring autophagosome formation or ULK1 phosphorylation) to isolate the precise causal node. In parallel, dissecting potential non-canonical telomerase functions, including mitochondrial TERT, redox protection, and interactions with FoxO and Wnt/β-catenin, will clarify whether telomerase has been evolutionarily repurposed as a coordinator of the autophagy–senescence axis in *Hydra*.

## Figures and Tables

**Table 1:** Summary and Categorization of Evidence Levels for the Proposed Integrative Model.

Interaction Node	Species/ Strain	Perturbation	Measured Endpoint(s)	Evidence Level
FoxO in stem cell maintenance	*H. vulgaris*	FoxO RNAi/ overexpression	Stem cell self-renewal, interstitial cell pool	Direct Functional (*Hydra*)
FoxO in microbiome composition	*H. vulgaris*	FoxO deficiency	Antimicrobial peptide expression, Microbiota composition	Direct Functional (*Hydra*)
Autophagy in homeostasis	*H. vulgaris*	WIPI2 / ULK1 RNAi; SBI-0206965	Body size, regeneration, aging-like phenotypes	Direct Functional (*Hydra*)
Starvation-induced autophagy	*H. vulgaris*	Starvation	Autophagosome formation, lysosomal activity	Direct Functional (*Hydra*)
Cold-stressed autophagy block	cold-sensitive *H. oligactis*	Cold exposure (10 °C)	p62/SQSTM1 accumulation, vacuolization	Correlative (*Hydra*)
Tissue extracts on ULK1 activity	cold-sensitive *H. oligactis*/ *H. vulgaris*	In vitro biochemical assay	In vitro human ULK1 kinase	Cross-Species Biochemical
TRPA1-mediated cell response	*H. vulgaris*	Cold exposure/ TRPA1 agonists	Nrf2/NF-kB translocation, SOD/NOS expression	Direct Functional (*Hydra*)
TRPM3-mediated heat response	*H. vulgaris*	Noxious heat/ TRPM3 agonists	Nitric Oxide (NO) production, HSP70 expression	Direct Functional (*Hydra*)
TRPA1-FoxO longevity control	*C. elegans* (inferred)	TRPA1 deletion/ thermal shift	Lifespan extension, FoxO translocation	Cross-Species Analogy

## Data Availability

No new data were created or analyzed in this study. Data sharing is not applicable to this article.

## Abbreviations

The following abbreviations are used in this manuscript:

ATG4	Autophagy related 4
cGMP	Cyclic guanosine monophosphate
EGCG	Epigallocatechin gallate
FoxO	Forkhead box O
GMP	Germline Multipotency Program
HoCS	Cold-sensitive (strains of) *Hydra oligactis*
HoCR	Cold-resistant (strains of) *Hydra oligactis*
HSP70	Heat shock protein 70
LC3	Microtubule-associated protein 1 light chain 3 |
NF-κB	Nuclear factor kappa-light-chain-enhancer of activated B cells
NO	Nitric oxide
NOS	Nitric oxide synthase
Nrf2	Nuclear factor erythroid 2-related factor 2
POT1	Protection of telomeres 1
RAP1	Repressor/activator protein 1
RNAi	RNA interference
ROS	Reactive oxygen species
SASP	Senescence-associated secretory phenotype
sGC	Soluble guanylyl cyclase
SA-β-gal	Senescence-associated β-galactosidase
SOD	Superoxide dismutase
SQSTM1	Sequestosome 1
TERT	Telomerase reverse transcriptase
TIN2	TERF1-interacting nuclear factor 2
TPP1	TIN2-interacting protein 1
TORC1	Target of rapamycin complex 1
TRF1	Telomeric repeat-binding factor 1
TRP	Transient Receptor Potential
TRPA	Transient Receptor Potential Ankyrin
TRPM	Transient Receptor Potential Melastatin
TRPVL	Transient Receptor Potential Vanilloid-Like
ULK1	Unc-51 like autophagy activating kinase 1
WIPI2	WD repeat domain phosphoinositide-interacting protein 2
